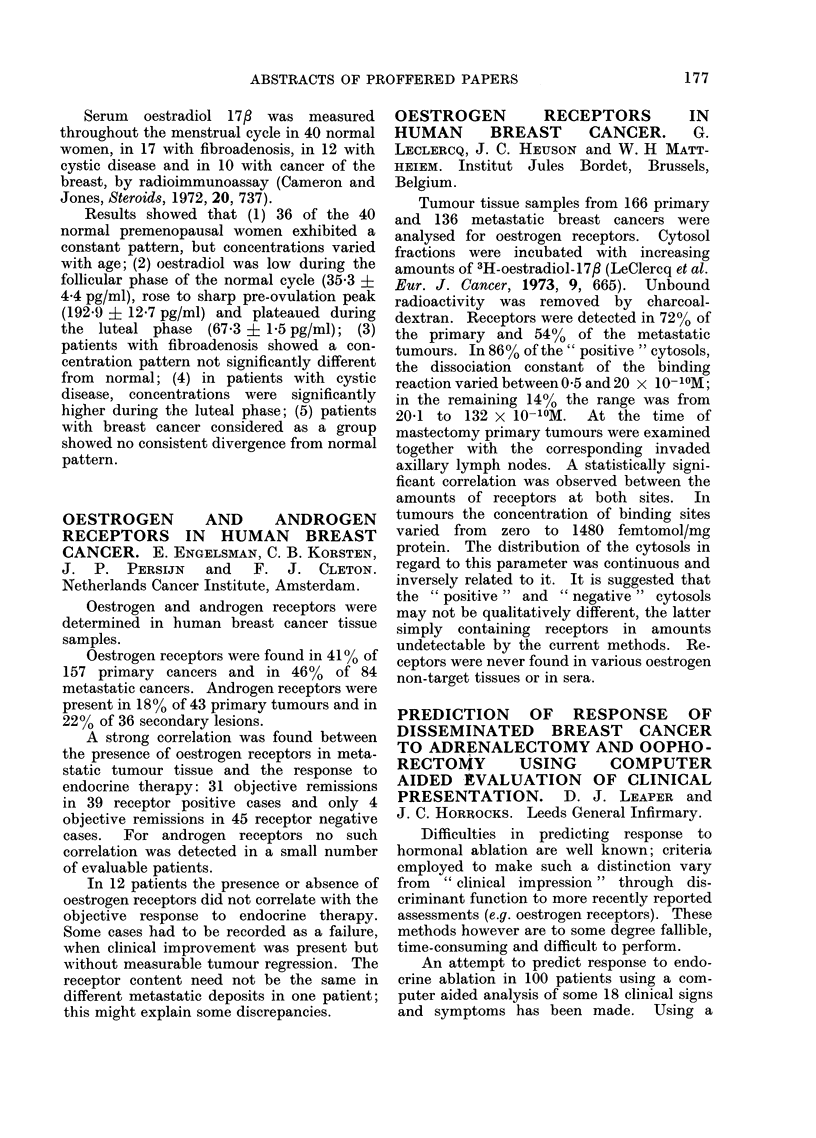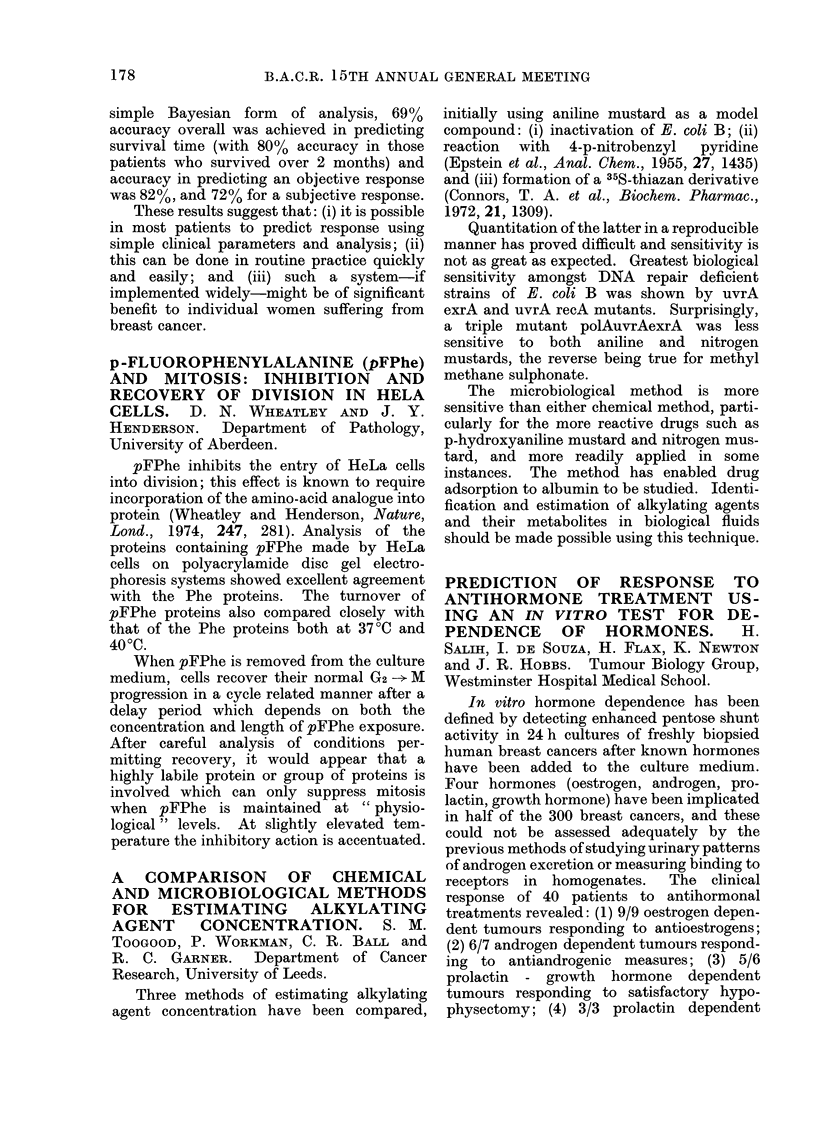# Proceedings: Prediction of response of disseminated breast cancer to andrenalectomy and oophorectomy using computer aided evaluation of clinical presentation.

**DOI:** 10.1038/bjc.1974.147

**Published:** 1974-08

**Authors:** D. J. Leaper, J. C. Horrocks


**PREDICTION OF RESPONSE OF DISSEMINATED BREAST CANCER TO ADRENALECTOMY AND OOPHORECTOMY USING COMPUTER AIDED EVALUATION OF CLINICAL PRESENTATION.** D. J. Leaper and J. C. Horrocks. Leeds General Infirmary.

Difficulties in predicting response to hormonal ablation are well known; criteria employed to make such a distinction vary from " clinical impression " through discriminant function to more recently reported assessments (*e.g.* oestrogen receptors). These methods however are to some degree fallible, time-consuming and difficult to perform.

An attempt to predict response to endocrine ablation in 100 patients using a computer aided analysis of some 18 clinical signs and symptoms has been made. Using a simple Bayesian form of analysis, 69% accuracy overall was achieved in predicting survival time (with 80% accuracy in those patients who survived over 2 months) and accuracy in predicting an objective response was 82%, and 72% for a subjective response.

These results suggest that: (i) it is possible in most patients to predict response using simple clinical parameters and analysis; (ii) this can be done in routine practice quickly and easily; and (iii) such a system—if implemented widely—might be of significant benefit to individual women suffering from breast cancer.